# The importance of Galectin-3 in the diagnosis of childhood acute appendicitis

**DOI:** 10.55730/1300-0144.6085

**Published:** 2025-09-15

**Authors:** Onur YALÇIN, Aybegüm KALYONCU AYÇENK, Ali AYGÜN, Ahmet Burak GÜRPINAR, Mehmet Seyfettin SARIBAŞ, Volkan ALTINOK, Tevfik NOYAN

**Affiliations:** 1 Department of Pediatric Surgery, Faculty of Medicine, Ordu University, Ordu, Turkiye; 2 Department of Emergency Medicine, Faculty of Medicine, Ordu University Ordu, Turkiye; 3 Department of Biochemistry, Faculty of Medicine, Ordu University, Ordu, Turkiye

**Keywords:** Acute appendicitis, diagnosis, Galectin-3, inflammation

## Abstract

**Background/aim:**

Acute appendicitis (AA) is a frequent indication for emergency surgery in children; accurate diagnosis is essential to prevent complications. We examined whether serum Galectin-3 is associated with pediatric AA and quantified its diagnostic performance relative to routine inflammatory markers.

**Materials and methods:**

This prospective, single-center study was conducted in a tertiary emergency department between July 1, 2023, and January 31, 2024. Children younger than 18 years with pathologically confirmed AA were enrolled as cases; age-compatible healthy volunteers served as controls. Venous blood was obtained at presentation before any therapeutic intervention. Galectin-3 concentrations were quantified using a commercial enzyme-linked immunosorbent assay. The primary endpoint was the ability of Galectin-3 to discriminate AA from controls. Receiver operating characteristic analysis was performed to assess discrimination, yielding area under the curve estimates with 95% confidence intervals (CIs); sensitivity and specificity were quantified at prespecified thresholds.

**Results:**

Seventy-four children were analyzed (AA group n = 47; control group n = 27). Compared with controls, the AA group had higher Galectin-3, leukocyte and neutrophil counts, C-reactive protein (CRP), neutrophil-to-lymphocyte ratio (NLR), platelet-to-lymphocyte ratio (PLR), and lower lymphocyte counts and platelet-to-neutrophil ratio (PNR) (all p < 0.05). Galectin-3 yielded an area under the curve (AUC) value of 0.680 (95% CI, 0.55–0.81). At a threshold of >130.71 pg/mL, sensitivity was 91.49% and specificity was 33.33%; at >115.71 pg/mL, sensitivity was 100.00% with the same specificity; at >254.90 pg/mL, specificity was 92.30% with sensitivity 29.79%.

**Conclusion:**

Serum Galectin-3 is elevated in pediatric AA and affords moderate discrimination. Owing to limited specificity at pragmatic cut-offs, Galectin-3 alone is insufficient as a standalone test; however, it may serve as a rule-out adjunct within multimodal pathways. External validation in larger, multicenter cohorts is warranted.

## 1. Introduction

Acute appendicitis (AA) represents one of the leading surgical diagnoses in emergency settings and is a prevalent reason for surgical intervention in patients presenting with acute abdominal pain [1,2]. Diagnosis is traditionally based on clinical symptoms, such as abdominal pain that initially localizes to the periumbilical region and then shifts to the right iliac fossa, often accompanied by nausea and peritoneal signs on physical examination [3,4]. Although various laboratory tests and imaging modalities are employed for rapid and accurate diagnosis, they have limitations in differentiating AA from other causes of abdominal pain [5]. In particular, diagnosing AA in children is more challenging because of difficulties in obtaining a reliable physical examination and the child’s limited ability to articulate their pain. History taking, detailed physical examination, elevated inflammatory parameters in blood tests, radiological findings, and clinical experience all play critical roles in diagnosis. Delayed diagnosis may result in complications such as appendiceal perforation, peritonitis, intraabdominal abscess, sepsis, ileus, and even death [6]. Ultrasonography (US) and computed tomography (CT) are frequently used to diagnose appendicitis but require specialized equipment and experienced radiologists [7,8]. Therefore, the diagnostic value of inflammatory markers in routine tests such as complete blood count and biochemical tests has begun to be investigated. Clinicians seek new approaches both to reduce malpractice claims and to lower the rate of negative appendectomies.

Galectins constitute a family of β-galactoside-binding lectins, with 15 members identified in mammals to date. These proteins are categorized into three structural types: prototype, tandem–repeat, and chimera. Among galectins, Galectin-3 is the sole representative of the chimera-type subgroup. It is an approximately 35-kDa protein encoded by LGALS3 on chromosome 14. Its N-terminal domain mediates oligomerization, is a substrate for matrix metalloproteinase-dependent cleavage, and engages a range of intracellular binding partners. Galectin-3 demonstrates extensive tissue distribution in humans and is notably enriched in immune-related cells, including macrophages, monocytes, eosinophils, natural killer cells, mast cells, as well as activated B and T lymphocytes [9–11].

Due to its capacity to be actively released by damaged or inflamed cells to the cell surface as well as into bodily fluids like serum and urine, Galectin-3 is considered a promising biomarker for both the diagnosis and prognosis of various pathological conditions. [12–14]. Multiple studies have identified Galectin-3 as an important biomarker with diagnostic and prognostic relevance across a range of conditions, including cardiovascular diseases, renal pathologies, autoimmune disorders, neurodegenerative conditions, and cancer development. [14–18].

In this study, we aimed to evaluate serum Galectin-3 levels in patients who were diagnosed with AA and to determine the role of Galectin-3 in the early and accurate diagnostic evaluation of AA. Additionally, our objective was to understand the correlation between Galectin-3 levels and the severity of inflammation to assess its diagnostic accuracy.

## 2. Materials and methods

### 2.1. Study design and selection of participants

This study was designed as a prospective method and conducted at a tertiary care training hospital between 01.07.2023 and 31.01.2024. Ethical approval for this study was obtained from Ordu University Clinical Research Ethics Committee with protocol number 2023/88.

During the specified period, patients aged < 18 years who presented to the pediatric emergency department or pediatric surgery outpatient clinic with complaints of abdominal pain, underwent surgery with a preliminary diagnosis of AA, and whose postoperative pathology results were consistent with AA, were included in the study as the case group. Healthy volunteer patients aged < 18 years who visited the hospital for general check-up purposes and had no known illnesses were included in the control group, provided that informed consent was obtained.

Patients who presented with trauma, those whose postoperative pathology was not consistent with AA, patients with complicated appendicitis, those who were found to have a nonappendicitis infectious focus upon evaluation, patients with missing data in their medical records, those with a medical history of conditions known to affect hemogram parameters (such as immune thrombocytopenic purpura, disseminated intravascular coagulation, sepsis-related thrombocytopenia, major hemorrhage, Wiskott–Aldrich syndrome, myeloproliferative disorders, leukemia, vasculitis, splenectomy, thrombocytopenia with absent radius syndrome, storage pool diseases, iron deficiency anemia, and megaloblastic anemia), and individuals lacking documented informed consent (patient or guardian) were not included in the study.

### 2.2. Data collection and assessment of laboratory parameters

Demographic data (age, sex) of patients who were eligible for inclusion and exclusion criteria, along with laboratory parameters including leukocyte count, hemoglobin level, red cell distribution width (RDW), platelet count, platelet distribution width (PDW), neutrophil and lymphocyte counts, C-reactive protein (CRP), and Galectin-3 levels, obtained from blood samples, were recorded. Derived ratios, including the neutrophil-to-lymphocyte ratio (NLR = Neutrophil/Lymphocyte count), platelet-to-lymphocyte ratio (PLR = Platelet/Lymphocyte count), platelet-to-neutrophil ratio (PNR = Platelet/Neutrophil count), and PDW/RDW ratios were calculated and documented.

All the patients were divided into two groups: the AA and the control groups. Venous blood samples were collected upon admission. All laboratory samples were obtained at presentation, before any medical or surgical interventions were initiated. Blood samples were drawn into serum-separator tubes up to the upper vacuum level. Serum samples were collected using gel tubes with separators, while potassium-ethylenediaminetetraacetic acid (EDTA) tubes were employed for complete blood count analysis. Following 10 min of centrifugation at 3000 rpm, the plasma was isolated and stored at −80 degrees until further analysis. Serum CRP levels were measured spectrophotometrically (Cobas 6000 series c501 modular analyzer, Roche Diagnostics, North America) in the hospital laboratory. Leukocyte counts were obtained using the XN–1000 Automated Hematology Analyzer (Sysmex Corporation, Kobe, Japan) in the hospital laboratory. Serum Galectin-3 levels were evaluated with an enzyme-linked immunosorbent assay (ELISA) kit, kit no.: 202306015 (Bioassay Technology Laboratory, BT lab, Shangai, China), following the manufacturer’s protocol. The concentrations of Galectin-3 in the samples were quantified and reported in picograms per milliliter (pg/mL).

### 2.3. Statistical analyses

All statistical evaluations were carried out using the MedCalc software package (version 20.009; Ostend, Belgium). Categorical variables appear as frequencies with proportions, whereas continuous variables are conveyed using the mean ± standard deviation (SD) and the median alongside the interquartile range (IQR, 25–75). Distributional checks for continuous variables relied on Shapiro–Wilk testing. Subsequent comparisons of two independent cohorts employed the Student t-test if appropriate, or the nonparametric Mann–Whitney U test otherwise.

To assess the diagnostic value of Galectin-3, leukocyte count, NLR, and CRP, receiver operating characteristic (ROC) curve analysis was applied. For Galectin-3 in particular, diagnostic indicators such as the area under the curve (AUC), sensitivity, and specificity were calculated. Statistical significance was accepted at a p-value <0.05.

## 3. Results

Fifty-five patients under 18 years presenting with abdominal pain to the pediatric emergency department or pediatric surgery clinics and operated on for suspected AA were considered. Eight did not meet analysis criteria—four due to normal postoperative pathology and four owing to incomplete records. The study included 47 patients for the case group (Group A) and 27 healthy volunteers for the control group (Group B). Baseline demographics (age, sex) are tabulated in Table 1.

**Figure 1:**
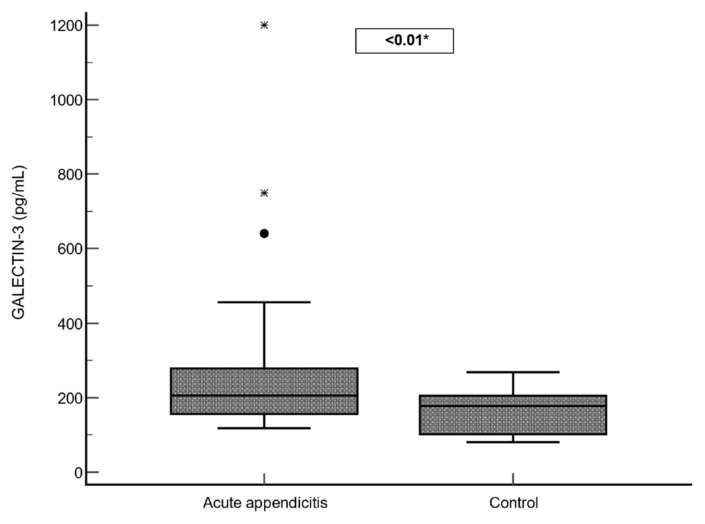
Box-plot graph of Galectin-3 values.

Compared with controls, the case group showed higher Galectin-3, leukocyte and neutrophil counts, CRP, PLR, and NLR, whereas lymphocyte counts and the PNR were lower (p < 0.05). In contrast, RDW, platelet count, PDW, and the PDW/RDW ratio did not differ significantly (p > 0.05). A box-plot comparison of Galectin-3 is presented in Figure 1. These results are summarized in Table 1.

**Figure 2:**
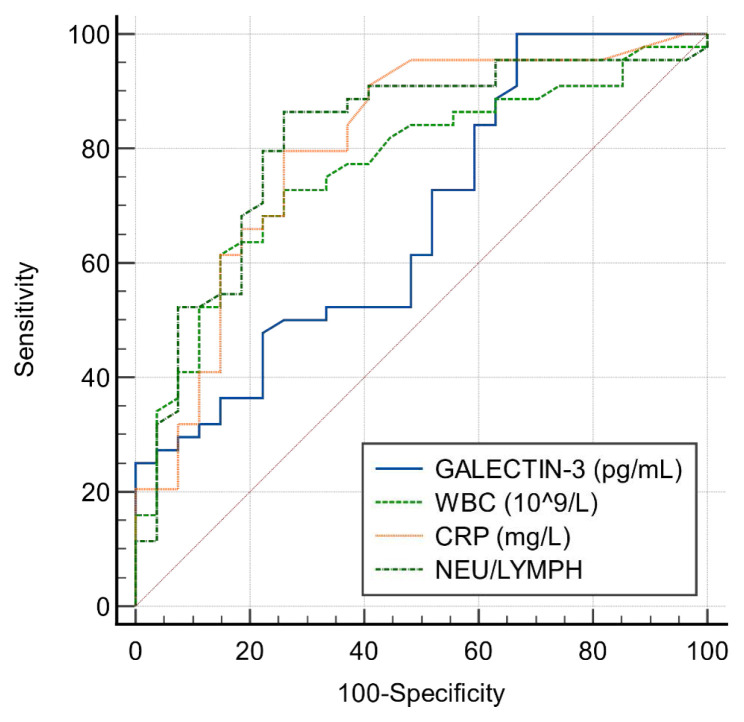
ROC analysis chart performed to measure the diagnostic value of hemogram parameters and Galectin-3 in patients with acute appendicitis.

To quantify diagnostic performance, we constructed ROC curves for serum Galectin-3; the area under the curve was 0.68 (95% CI 0.55–0.81; p < 0.05; Figure 2). Sensitivity and specificity at prespecified cut points are presented in Table 2. Higher thresholds were associated with greater specificity for pediatric appendicitis.

## 4. Discussion

Diagnosing AA in pediatric patients may be challenging due to the frequency of nonsurgical conditions in the differential diagnosis and difficulties in communication with children [19]. Classic clinical findings in the diagnosis of AA include fever, right lower quadrant pain starting from the periumbilical region, nausea, and vomiting, as well as biochemical markers such as white blood cell count, neutrophil count, CRP, and imaging modalities such as US and CT. However, all these parameters have certain limitations [20]. Becker et al. published in their study that many of the classical findings of AA were absent in pediatric patients diagnosed with the condition [21]. Although US is commonly used in children for the diagnosis of AA, it has disadvantages such as being operator-dependent and having highly variable sensitivity across different studies [19,22]. CT, with its high sensitivity and specificity, is often considered the gold standard for diagnosing AA. However, the exposure to ionizing radiation and associated concerns regarding increased cancer risk in pediatric populations have led to a consensus against using CT as the first-line diagnostic tool for AA [19,23]. Inflammation is crucial in the pathophysiology of AA. In the pathogenesis, secretion of cytokines, chemokines, and proinflammatory mediators contribute to the migration of inflammatory cells such as macrophages and neutrophils to the site of injury [24]. Many inflammatory biomarkers have been investigated for their potential to aid in the diagnosis and assessment of AA severity. A major advantage of inflammatory markers is that they are cost-effective and easy to access. It has been shown that biomarkers such as leukocyte count, NLR, PLR, and CRP can be useful in the diagnosis of AA [25–27]. In this study, consistent with the literature, elevated leukocyte count, NLR, PLR, and CRP levels were shown to be significantly associated with AA. These markers were significantly higher in the patients in the case group compared with the control group.

Galectins play roles in inflammatory processes such as cell differentiation, host defense against pathogens, and regulation of immune responses [28]. Galectin-3, present in diverse tissues (e.g., inflammatory cells and epithelial lineages), is implicated in core biological programs encompassing proliferation and differentiation, inflammatory activity, fibrogenesis, and immune system regulation [29]. Galectin-3 contributes to the initiation of inflammation by inducing macrophage chemotaxis. It also plays a role in sustaining the inflammatory response through the release of proinflammatory cytokines [30]. Galectin-3 release has been shown to increase in response to both microbial and nonmicrobial inflammatory stimuli. In infectious settings, Galectin-3 appears to participate in pathogen recognition while also serving as a marker of tissue injury. Multiple studies have reported that viral, bacterial, and fungal infections are associated with elevated Galectin-3 concentrations, and that higher levels may correlate with infection severity and prognostic outcomes [31–33].

Given the role of Galectin-3 in inflammatory processes, its utility as a biomarker has been investigated in several pathological conditions. Markovic et al. demonstrated in an experimental rat model that Galectin-3 is involved in the development of acute colitis and exhibits a proinflammatory response during the induction phase [34]. Li et al. also reported that Galectin-3 is an important marker for predicting sepsis-associated kidney injury [35]. During the Coronavirus disease 2019 (COVID-19) pandemic, Kartal Baykan et al. examined the role of Galectin-3 in the diagnosis of COVID-19 pneumonia and found that Galectin-3 was useful not only in diagnosis but also in predicting disease severity [36]. To date, no studies investigating the role of Galectin-3 in the diagnosis of AA have been encountered in the literature. This work contributes substantive, incremental insight to the literature. In the present study, elevated Galectin-3 levels successfully predicted AA in pediatric patients, with an AUC of 0.680. In this pediatric population, when the Galectin-3 cut-off value was set at >115.71 pg/mL, sensitivity reached 100% with a specificity of 33.33%; at a cut-off value of >130.71 pg/mL, sensitivity was 91.49% with the same specificity of 33.33%. Considering the role of inflammation in the pathogenesis of AA and the involvement of Galectin-3 in inflammatory processes, the elevated Galectin-3 levels observed in pediatric patients with AA appear to be explainable.

In our cohort, Galectin-3 demonstrated high sensitivity but low specificity. This profile indicates that while Galectin-3 is unlikely to miss true appendicitis cases (low false-negative rate), it may generate a considerable number of false positives, limiting its stand-alone diagnostic value. Clinically, such a biomarker is better positioned as a rule-out tool: a low Galectin-3 level at presentation could support conservative management or observation in low-risk children, thereby avoiding unnecessary imaging or surgery. Conversely, elevated Galectin-3 should not be interpreted in isolation but rather in combination with conventional inflammatory markers such as CRP, WBC, and NLR, and integrated into US-first diagnostic pathways. In this context, Galectin-3 may contribute to reducing CT utilization, refining risk stratification, and improving patient safety.

In a metaanalysis of 67 pediatric appendicitis studies, Fawkner-Corbett et al. showed that WBC and ANC were the most discriminative blood tests for rule-out, and that combining WBC with CRP improved diagnostic accuracy [37]. Another metaanalysis (19 studies; 5974 children) found NLR had moderate-to-good accuracy (sensitivity 0.82, specificity 0.76; AUC 0.86) for pediatric acute appendicitis [38]. In this context, our finding (Galectin-3 AUC ≈0.68) suggests Galectin-3 is suboptimal as a stand-alone test but may provide incremental value within multimarker algorithms.

The single-center nature of the study, coupled with a small sample, restricts the strength of inference and may limit applicability to wider pediatric populations. Larger, multicenter studies are therefore required to confirm these results. In addition, the imbalance in gender distribution between the case and control groups may have introduced potential bias, as sex-related biological differences could influence circulating biomarker levels such as Galectin-3. In the present study, healthy children served as controls. The use of healthy controls in diagnostic accuracy research can introduce spectrum bias, potentially limiting the extent to which specificity estimates reflect real-world clinical performance. Including children with abdominal pain due to nonappendicitis conditions would have allowed a more clinically relevant appraisal of Galectin-3 specificity. In addition, because complicated appendicitis can substantially affect inflammatory biomarker levels, patients with complicated disease were excluded from the study. Finally, Galectin-3 testing is presently relatively costly and not uniformly available. We did not perform a formal cost-effectiveness analysis, and test availability/turnaround may vary across institutions, which may constrain real-world implementation and should be considered when interpreting the applicability of our findings.

Our findings indicate that Galectin-3 levels are significantly elevated in patients diagnosed with AA and that this biomarker may possess a certain diagnostic value in predicting AA. However, due to its low specificity, Galectin-3 alone may not serve as a reliable diagnostic tool. It is suggested that Galectin-3 may offer additional diagnostic value when used alongside existing biomarkers in the diagnosis of AA.

## Figures and Tables

**Table 1:** Comparison of demographics. Hemogram and Galectin-3 values of acute appendicitis and control groups.

	Acute appendicitis group (n = 47)	Control group (n = 27)	p value

**Age (year)**	12.64 ± 3.57	11.89 ± 2.86	0.578
* **Female**	24 (51.1%)	5 (18.5%)	
* **Male**	23 (48.9%)	22 (81.5%)	

**Galectin-3 (pg/mL)**	205.88 (156.13–278.68)	177.45 (101.79–205.15)	<0.001

**WBC (10^9/L)**	14.3 (10.35–19.08)	9.2 (7.05–11.43)	<0.001

**Neutrophils (10^9/L)**	11.7 (7.45–16.78)	3.7 (2.33–6.2)	<0.001

**Lymphocytes (10^9/L)**	1.8 (1.2–2.4)	3.1 (2.43–4.23)	<0.001

**CRP (mg/L)**	34.05 (2.8–95.95)	0.8 (0.4–4.98)	<0.001

**RDW (%)**	12.9 (12.35–13.28)	13.2 (12.55–14.6)	0.080

**Platelets (10^9/L)**	301 (229–354)	311 (251.75–364.25)	0.536

**MPV (fL)**	9.5 (9.1–10)	9.7 (9.1–10.35)	0.582

**PDW (fL)**	10.3 (9.6–11.08)	10.3 (9.53–11.88)	0.578

**NLR**	7.6 (2.9–10.78)	1 (0.63–2.53)	<0.001

**PNR**	24.8 (20.1–41.28)	93.4 (46.68–127.95)	<0.001

**PLR**	165.3 (110.03–217.4)	94.5 (75.48–121.88)	<0.001

**PDW/RDW**	0.8 (0.7–0.9)	0.8 (0.7–0.9)	0.702

Results presented as n (%), mean ± SD, and median with interquartile range (25th–75th percentile).

CRP: C-Reactive protein. MPV: Mean platelet volume. NLR: Neutrophil-to-lymphocyte ratio. PDW: Platelet Distribution width. PLR: Platelet to lymphocyte ratio. PNR: Platelet to neutrophils ratio. RDW: Red cell distribution width. WBC: White blood cell count.

* Chi-square test.

Variables are expressed as mean ± standard deviation or n (%).

**Table 2:** The Performance characteristics of Galectin-3 according to cut-off values in predicting acute appendicitis.

Galectin-3 (pg/mL)	Sensitivity	Specificity	+LR	−LR	PPV (%)	NPV (%)
	(%95 CI)	(%95 CI)				
>115.71	100 (92.5–100)	33.33 (16.5–54)	1.5	0.0	72.3	100
>130.71	91.49 (79.6–97.6)	33.33 (16.5–54.0)	1.37	0.26	70.5	69.2
>157.84	72.34 (57.4–84.4)	48.15 (28.7–68.1)	1.40	0.57	70.8	50
>254.90	29.79 (17.3–44.9)	92.30 (81.0–99.9)	8.040	0.73	93.3	44.1

CI: Confidence interval. LR: Likelihood ratio. NPV: Negative predictive value. PPV: Positive predictive value. ROC: Receiving operating characteristics.
